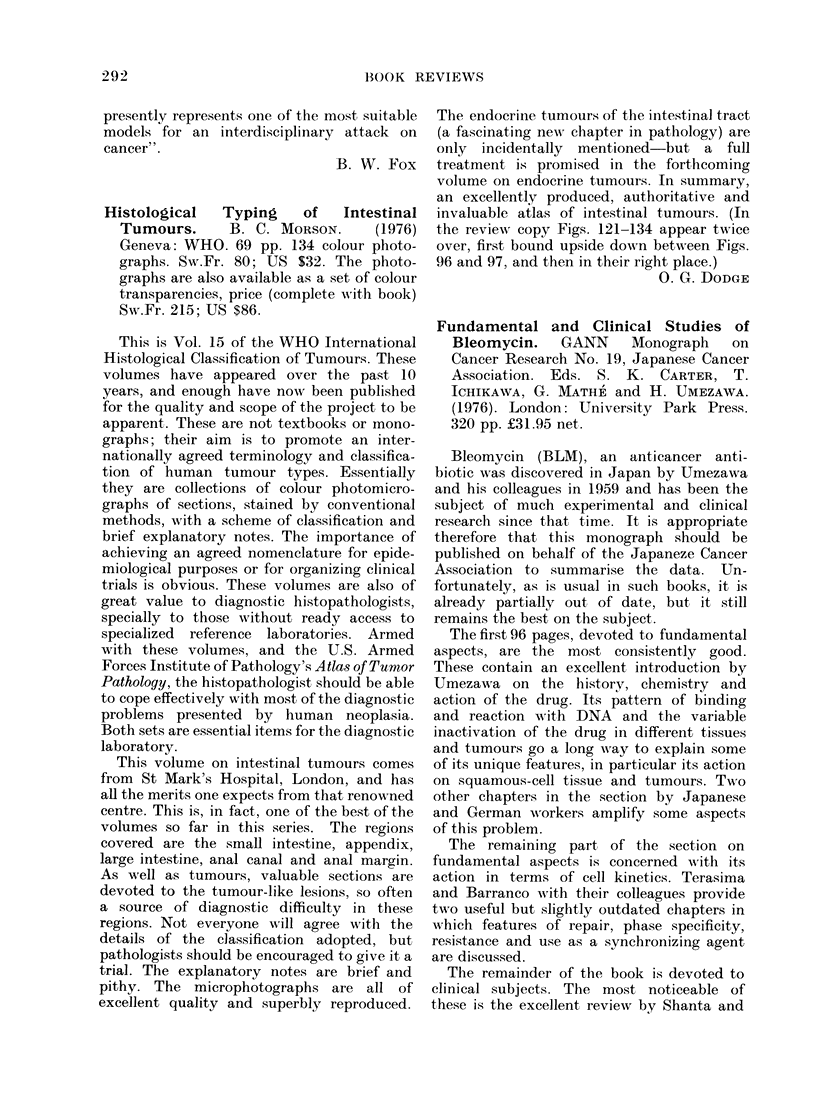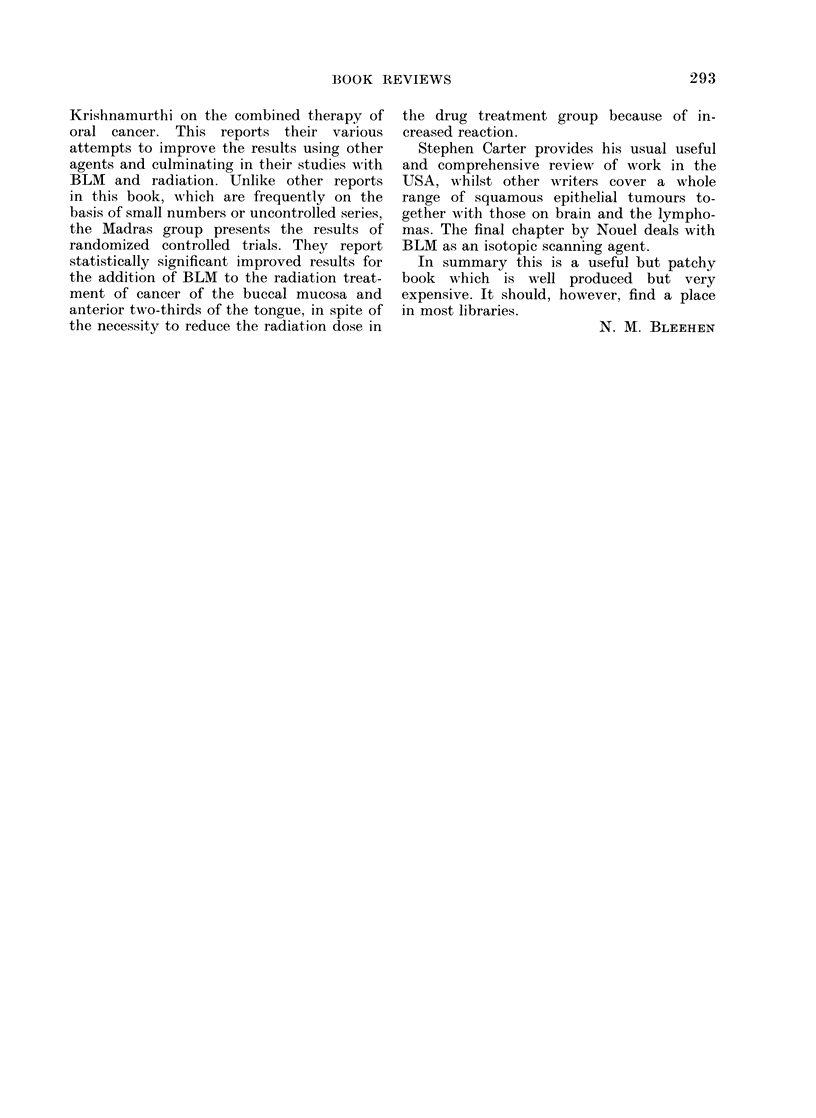# Fundamental and Clinical Studies of Bleomycin

**Published:** 1977-08

**Authors:** N. M. Bleehen


**Fundamental and Clinical Studies of Bleomycin.** GANN Monograph on Cancer Research No. 19, Japanese Cancer Association. Eds. S. K. Carter, T. Ichikawa, G. Mathé and H. Umezawa. (1976). London: University Park Press. 320 pp. £31.95 net.

Bleomycin (BLM), an anticancer antibiotic was discovered in Japan by Umezawa and his colleagues in 1959 and has been the subject of much experimental and clinical research since that time. It is appropriate therefore that this monograph should be published on behalf of the Japaneze Cancer Association to summarise the data. Unfortunately, as is usual in such books, it is already partially out of date, but it still remains the best on the subject.

The first 96 pages, devoted to fundamental aspects, are the most consistently good. These contain an excellent introduction by Umezawa on the history, chemistry and action of the drug. Its pattern of binding and reaction with DNA and the variable inactivation of the drug in different tissues and tumours go a long way to explain some of its unique features, in particular its action on squamous-cell tissue and tumours. Two other chapters in the section by Japanese and German workers amplify some aspects of this problem.

The remaining part of the section on fundamental aspects is concerned with its action in terms of cell kinetics. Terasima and Barranco with their colleagues provide two useful but slightly outdated chapters in which features of repair, phase specificity, resistance and use as a synchronizing agent are discussed.

The remainder of the book is devoted to clinical subjects. The most noticeable of these is the excellent review by Shanta and

Krishnamurthi on the combined therapy of oral cancer. This reports their various attempts to improve the results using other agents and culminating in their studies with BLM and radiation. Unlike other reports in this book, which are frequently on the basis of small numbers or uncontrolled series, the Madras group presents the results of randomized controlled trials. They report statistically significant improved results for the addition of BLM to the radiation treatment of cancer of the buccal mucosa and anterior two-thirds of the tongue, in spite of the necessity to reduce the radiation dose in the drug treatment group because of increased reaction.

Stephen Carter provides his usual useful and comprehensive review of work in the USA, whilst other writers cover a whole range of squamous epithelial tumours together with those on brain and the lymphomas. The final chapter by Nouel deals with BLM as an isotopic scanning agent.

In summary this is a useful but patchy book which is well produced but very expensive. It should, however, find a place in most libraries.

N. M. Bleehen